# Percutaneous mitral valve repair: The MitraClip device

**DOI:** 10.21542/gcsp.2016.17

**Published:** 2016-06-30

**Authors:** Hussam S. Suradi, Clifford J. Kavinsky, Ziyad M. Hijazi

**Affiliations:** 1 St Mary Medical Center, Community HealthCare Network, Hobart, IN; 2 Rush Center for Structural Heart Disease, Rush University Medical Center, Chicago, IL; 3 Sidra Cardiovascular Center of Excellence, Sidra Medical & Research Center, Doha, Qatar

## Abstract

Chronic mitral regurgitation (MR) is the most common cardiac valvular disease with more than 4 million people in the United States alone suffering from moderate or severe MR. Left untreated, chronic MR results in serious consequences. Surgical correction with mitral valve repair or replacement remains the mainstay of therapy for MR. Nevertheless, a large proportion of patients may not be offered treatment due to concerns over surgical risk. This unmet therapeutic need for a less invasive approach led to a marked explosion in the development of a variety of transcatheter approaches to treat mitral regurgitation in the past decade. The majority of these devices are based on principles learned from surgical mitral valve repair techniques. Inspired by the Alfieri surgical technique, the MitraClip edge-to-edge repair system is the most advanced technique with the highest safety and efficacy to date. In this review, we discuss the current status of the MitraClip repair system in the treatment of mitral regurgitation.

## Introduction

Chronic mitral regurgitation (MR) poses a significant public health burden with more than 4 million people in the United States suffering from moderate or severe MR with nearly 250,000 new diagnoses of MR made each year^1,2^. Left untreated, chronic severe MR leads to left ventricular remodeling, myocardial dysfunction, heart failure, left atrial dilatation, atrial fibrillation and pulmonary hypertension. Medical therapy may alleviate symptoms; however, there is no proven benefit to medical therapy in terms of treating the underlying pathophysiology of MR or retarding its progression^3^. Therefore, surgical valve repair or replacement remains the standard of care for treating patients with chronic MR. Nevertheless, it is estimated that only about 20% of patients with significant MR are offered surgery with a large proportion of patients left untreated due to unavailability of surgical treatment or concerns about surgical risk, specifically those with advanced age and impaired left ventricular function. Therefore, there is a clear unmet need for patients with MR for a less invasive therapeutic alternative.

The mitral valve is a complex structure that consists of several integrated components. This functional unit includes the mitral annulus, mitral leaflets, the subvalvular apparatus (papillary muscles and chordae tendineae), the left atrium and left ventricular myocardium. The mitral leaflets consist of a larger anterior leaflet and a smaller posterior leaflet, each subdivided into three scallops (lateral, middle and medial scallops). Each leaflet is attached to the annulus and is connected to papillary muscles by a web of chordae tendineae. This subvalvular apparatus prevents the leaflets from prolapsing into the left atrium and is essential in maintaining ventricular shape and contractility. These components of the mitral apparatus are integral to the normal function of the valve and each is a potential venue for repair.

**Figure 1.**
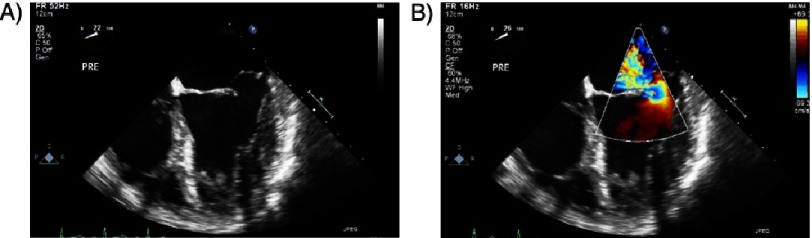
(A–B) Severe degenerative MR due to posterior mitral leaflet prolapse involving the middle scallop.

**Figure 2.**
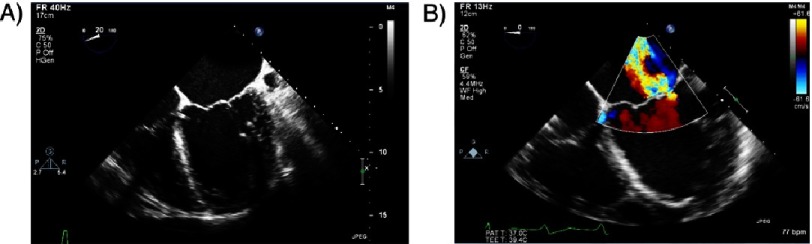
(A–B) Severe functional MR in a patient with severe cardiomyopathy.

In addressing the current therapy of MR, it is useful to distinguish degenerative (primary) from functional (secondary) MR (Figures 1 and 2). In degenerative MR, abnormalities of one or more of the components of the mitral valve cause it to leak such as myxomatous degeneration leading to leaflet prolapse or chordal rupture. On the other hand, functional MR is caused by geometric remodeling of the left ventricle without structural abnormalities of the leaflets or chordae. This occurs in patients with dilated and ischemic cardiomyopathy causing papillary muscle displacement and annular dilatation resulting in malcoaptation of the leaflets.

**Figure 3.**
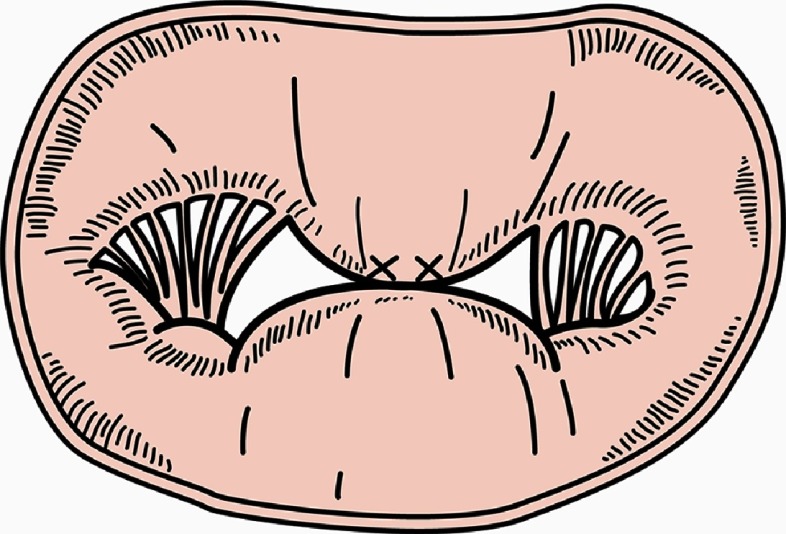
Alfieri surgical repair technique.

In 1991, Alfieri and his colleagues described the surgical end-to-end mitral valve repair, whereby the leading edges of central scallops of the mitral leaflets are sutured together at the origin of MR, creating a double-orifice mitral valve^4^ (Figure 3). Success and simplicity of this technique inspired the development of a catheter-based technology that would enable a less invasive percutaneous valve repair. The MitraClip (Abbott Laboratories, Abbott Park, IL) evolved as the world’s first transcatheter mitral valve repair method to create an edge-to-edge mitral valve repair using a transseptal approach. The first implantation in a human was in 2003 in Venezuela^5^. The device subsequently achieved CE mark approval in 2008 in Europe and FDA approval in 2013 in the United States.

This review discusses the percutaneous edge-to-edge mitral valve repair using the MitraClip system for the management of mitral regurgitation.

## The MitraClip system

**Figure 4.**
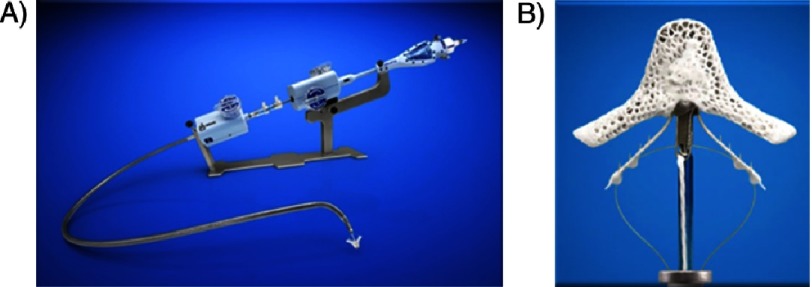
(A) MitraClip device and (B) clip (Courtesy of Abbott).

Inspired by the Alfieri surgical repair, the MitraClip device utilizes a catheter-delivered clip rather than a suture to create a double orifice in an attempt to reduce the regurgitation jet. The MitraClip system consists of a steerable guide catheter and a clip delivery system (CDS) (Figure 4). The guide catheter is 24 French proximally and tapers to 22 French at the point where it crosses the atrial septum. A steering knob on the proximal end of the guide catheter allows controlled deflection of the distal tip. The clip delivery system (CDS) includes the detachable clip. This system is steerable using two knobs that permit medial-lateral and anterior-posterior steering. The clip is a dacron-covered implant with two arms that are opened and closed by a control mechanism on the CDS. The two arms have a span of approximately 2 cm when opened in the grasping position; the width of the clip is 4 mm. On the inner portion of the clip are two “grippers”. Each gripper matches up to each arm and helps to stabilize the leaflets from the atrial aspect as they are captured during closure of the clip arms. The clip arms are covered with polyester fabric to promote tissue growth.

## Procedural technique

**Figure 5.**
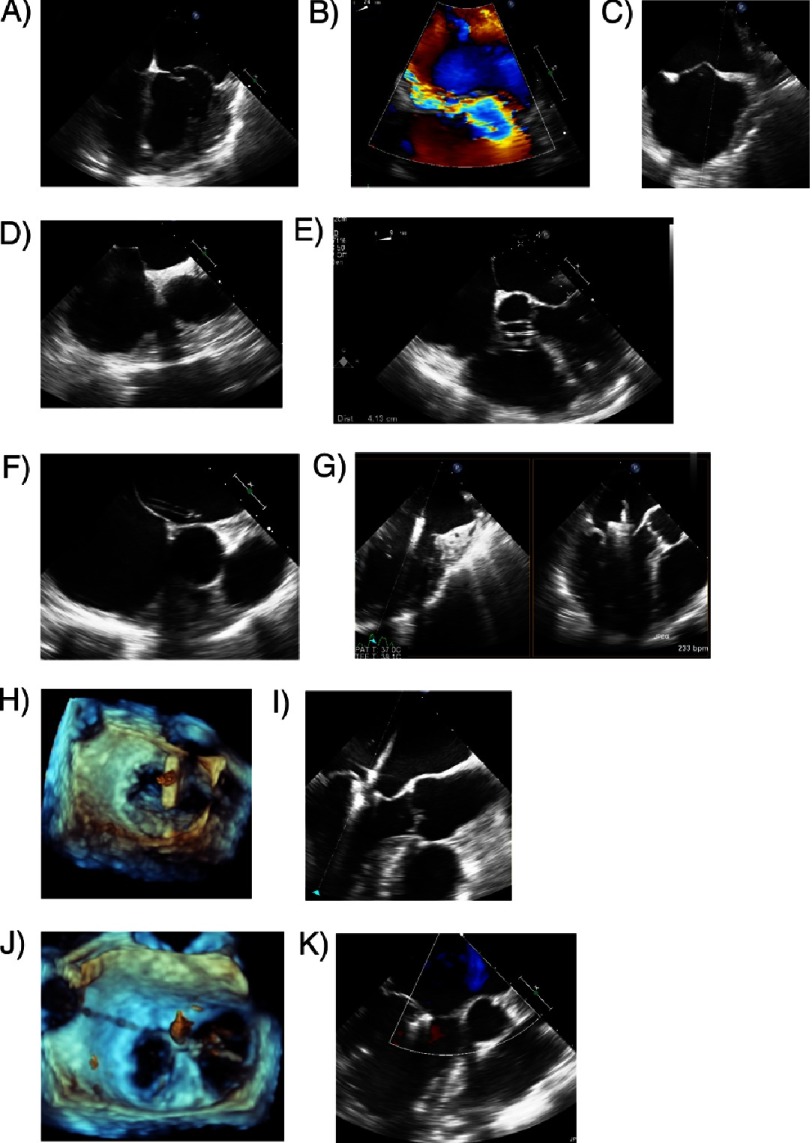
Echocardiographic steps of the procedure. (A–B) Severe MR due to flail portion of the posterior middle scallop (P2). (C–E) Transseptal puncture. Using transesophageal echo bi-caval (C) and short axis (D) views, position of the septal tenting is evaluated with puncture site performed at the posterosuperior aspect of the septum. Afterward, using four-chamber view (E), the catheter tip should be at least 3 cm above the plane of the mitral valve. (F) Advancement of sheath into the left atrium. (G) The device is steered until the trajectory is perpendicular to the line between the annular hinge points of the mitral valve in both the left ventricular outflow tract (anteroposterior alignment) and two-chamber intercommissural (mediolateral alignment) imaging views. (H) En-face view to check perpendicularity of the clip to the mitral leaflets. (I) The leaflets are grasped by retracting the delivery catheter slowly as the mitral leaflets are closing in systole in the LVOT view. (J–K) Final result with double-orifice appearance of the mitral valve with no residual MR.

**Figure 6.**
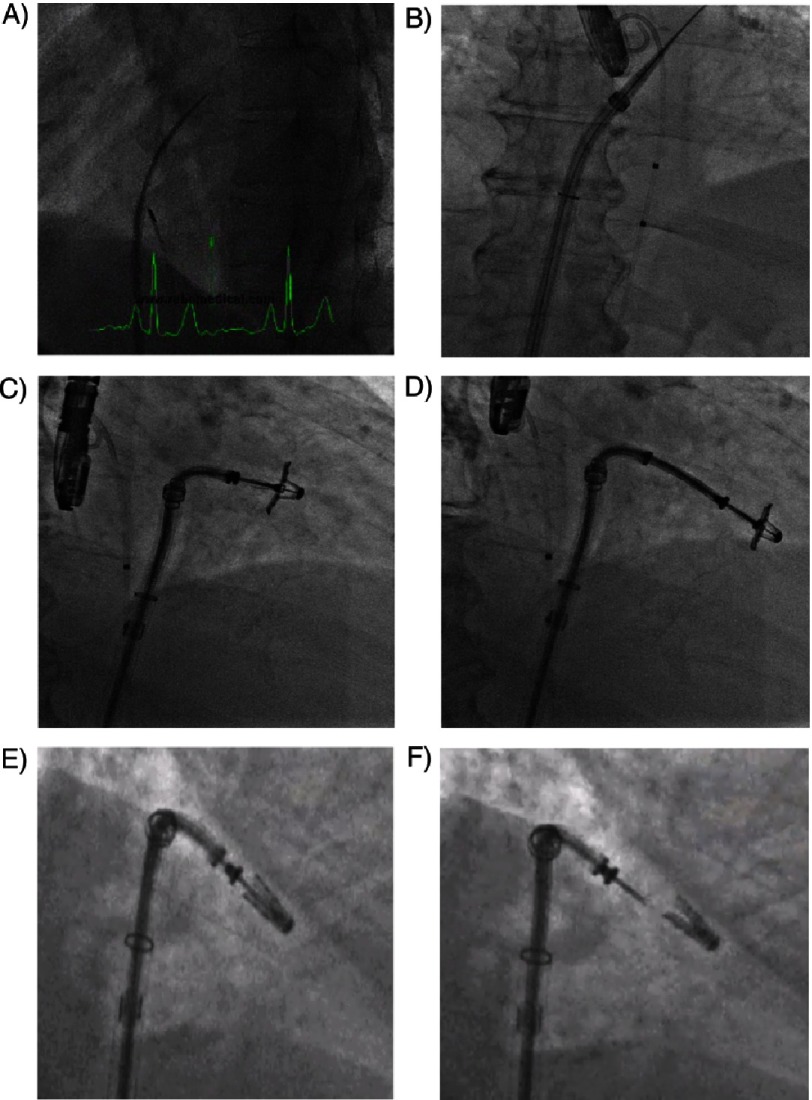
Fluoroscopic steps of the procedure. (A) Atrial septal puncture. (B) Guide catheter advanced into left atrium. (C) Clip with open arms advanced into the left atrium towards the mitral valve. (D) MitraClip with opened arms is advanced into the left ventricle. (E) Leaflets are grasped and clip is closed. (F) Clip is released.

The MitraClip repair is performed under general anesthesia in the cardiac catheterization laboratory with fluoroscopy and transesophageal imaging guidance (two and three-dimensional imaging). The procedure consists of four main steps (Figures 5 and 6):

(1) Vascular access, transseptal puncture and steerable guide catheter insertion.
(2) Steering of the guide catheter and clip delivery system (CDS) into the left ventricle.
(3) Leaflet grasping and clip closure.
(4) Clip deployment and system removal.

After right heart catheterization is performed, transseptal puncture is performed under TEE guidance. This is a critical step of the procedure as the puncture site should be located in the posteroior-superior part of the interatrial septum, 3 to 4 cm above the plane of coaptation of the mitral valve leaflets. This is of course done under TEE guidance in two orthogonal views: the short axis that will determine anterior-posterior positions and in the long axis view that will determine superior-inferior relationship of the puncture. The puncture site should be posterior-superior location that allows adequate room in the left atrium for optimal orientation of the steerable CDS. Once the left atrium is entered with the Mullins sheath, unfractionated heparin is administered maintaining an ACT >250 seconds throughout the procedure. The left pulmonary vein is then cannulated using a multipurpose catheter and a 260 cm Amplatz Super stiff guidewire is left in place. The Mullins sheath is then exchanged for the 24 F guiding catheter and introduced into the left atrium over the guidewire. The dilator is then slowly and carefully retrieved to avoid air embolism. The clip delivery system, with a clip attached to its distal end, is then advanced into the left atrium, and the distal steerable part is manipulated in the atrium for obtaining a perpendicular and central position with respect to the mitral valve leaflets’ coaptation line. Under transesophageal echocardiographic guidance, the clip is steered until axially aligned and centered over the origin of the regurgitant jet. The clip is opened and the grippers are retracted. The correct trajectory of the clip and the perpendicularity of the two arms with respect to the mitral leaflet coaptation line are checked using echocardiography.

Once the system has been aligned, the clip with opened arms is advanced into the left ventricle and under transesophageal echocardiographic (TEE) guidance the arms grasp the leaflets and the grippers are lowered such that the clip is partially closed to secure the leaflets. Stable grasp of both leaflets and MR reduction are carefully assessed using TEE. If the position is judged suboptimal by TEE evaluation, the clip can be reopened and repositioned. In the event that the clip must be withdrawn back into the left atrium, the arms can be inverted in the left ventricle and pulled back into the left atrium, avoiding entanglement of the clip with the chordae tendineae. If reduction of MR is deemed adequate and there is no significant mitral stenosis (transmitral gradient ≤ 5 mmHg), the clip is deployed and the CDS is withdrawn. If significant residual MR is noted, a second clip can be implanted adjacent to the first clip at the location of the residual MR. Following final echocardiographic and hemodynamic assessment, the guide catheter is then removed and femoral venous access is closed as per operator preference. We favor using a “figure-of-eight” stitch.

Post-procedural pharmacologic management includes aspirin 81 mg lifelong and clopidogrel 75 mg for three months in patients without atrial fibrillation. Patients with atrial fibrillation are generally prescribed aspirin and coumadin.

## Complications

The MitraClip repair is associated with an overall complication rate of 15–19% at 30 days^6,7^. Early complication events are primarily related due to need for periprocedural blood transfusion, while late events are primarily related to underlying heart failure or patient comorbidities.

Access site bleeding is a potential complication due to the large venous sheath size. In the EVEREST II trial, 13 percent of patients randomized to MitraClip required transfusion of ≥2 units of blood, compared to 45 percent of patients who underwent surgical mitral valve repair. In registries of MitraClip, the percentage of patients requiring ≥2 units of blood transfusion has been significantly lower, ranging from 0.9 to 3.9 percent^8,9^. Other potential procedural complications include those secondary to transseptal puncture and air embolism. Therefore, careful echocardiographic guidance as well as careful aspiration and flushing of the steerable guide are essential to avoid such complications.

Device embolization is an exceedingly rare complication. More commonly, partial clip detachment, defined as detachment of a single leaflet from the clip, may occur and requires surgical treatment. In EVEREST II study, 9 patients had partial clip detachment in the first 12 months, one additional patient developed partial clip detachment from years one to four. All patients subsequently underwent mitral valve surgery^6^.

The development of clinically significant mitral stenosis is another potential complication of MitraClip implantation that tends to be poorly tolerated by patients. Careful hemodynamic assessment prior to clip release is important to avoid such complication.

## Patient selection

Multidisciplinary team approach comprising an interventional cardiologist, cardiac surgeon, and echocardiographer working together is essential for the proper selection of candidates for the transcatheter mitral valve repair.

To establish clinical indication criteria, all patients undergo a standardized echocardiographic assessment protocol. Patients are required to meet basic criteria for intervention from the 2014 American College of Cardiology/American Heart association guidelines for valvular heart disease^10^. Currently, the MitraClip device is FDA approved in the US for patients with significant symptomatic primary (degenerative) MR who have been determined to be at prohibitive risk for mitral valve surgery by a heart team. Although MitraClip has been used to treat functional MR, no randomized controlled studies focusing on functional MR have been published to date. The COAPT trial (Clinical Outcomes Assessment of the MitraClip Percutaneous Therapy for High Surgical Risk), which is now underway in multiple sites, will be the first randomized controlled trial to evaluate the safety and efficacy of the use of the MitraClip device in patients with functional MR (see below).

**Figure 7.**
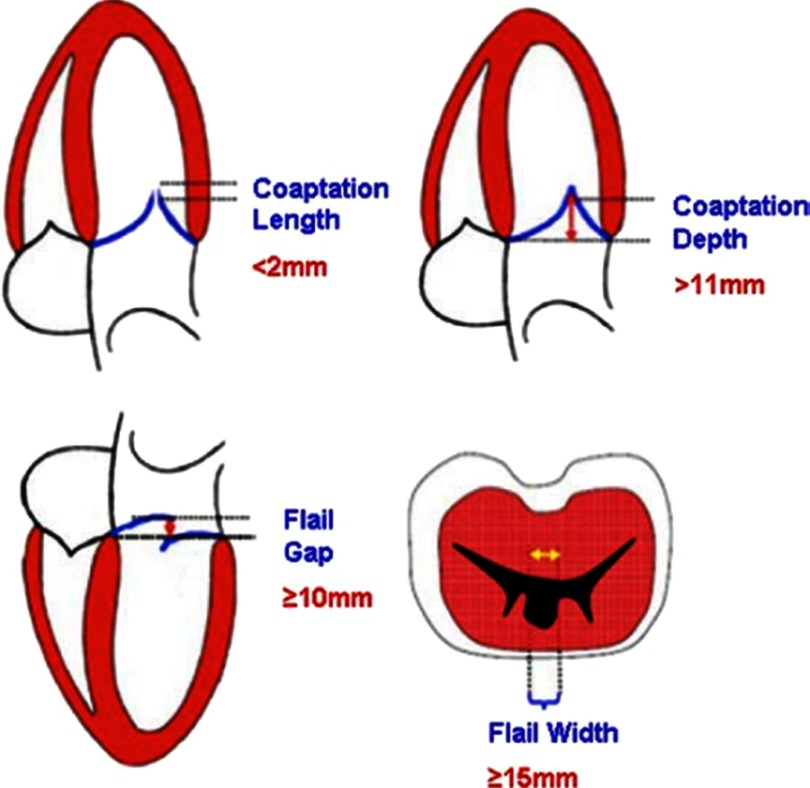
Key anatomical exclusion criteria for MitraClip.

In addition to meeting clinical indications, patients have to fulfill several anatomical criteria to qualify for MitraClip therapy (Figure 7). As discussed above, careful echocardiographic evaluation of the mitral leaflets to determine the exact mechanism of MR is critical for proper patient selection and exclusion of mitral valve pathologies (ie, rheumatic) that may not be amenable for transcatheter repair. Both degenerative and functional MR has been successfully treated using the MitraClip device.

To achieve optimal results in patients with a flail leaflet, the flail gap should not be greater than 10 mm with a flail width less than 15 mm on short-axis estimation. For patients with functional MR, leaflets coaptation length of at least 2 mm and coaptation length less than 11 mm are required in order to have adequate tissue to grasp with the clip. Leaflets with less coaptation length can possibly be grasped as well; however, data on long term outcome in this setting is limited. Furthermore, leaflets should be free of significant calcification in the grasping area. In addition, careful assessment of the subvalvular apparatus is needed and the presence of chordal tissue in excess may render orientation, recovery and clip stability cumbersome. It is also important to note that baseline mitral valve area should be greater than 4 cm^2^ to avoid the creation of mitral stenosis after clip deployment. For functional MR, it is important to determine whether the patient with ischemic cardiomyopathy has a need for concomitant revascularization. Lastly, patients with hemodynamic instability are unlikely to benefit solely from correction of their MR nor would patients who are truly in end-stage heart failure.

## MitraClip trial outcomes

The first successful human MitraClip implant was performed in 2003 in a 56 year old woman in Venezuela with reduction of severe mitral regurgitation due to bileaflet prolapse to <2+ with concomitant clinical improvement^5^. After this first procedure, the MitraClip approach to percutaneous mitral valve repair was evaluated in the United States in the Endovascular Valve Edge-to-Edge Repair Study (EVEREST) trials.

## EVEREST Trials

EVEREST I trial was a prospective, multicenter, phase I study to evaluate the safety and feasibility of the MitraClip procedure^11^. This represented the initial experience with this novel technology that has a steep learning curve. The study enrolled a total of 107 patients with moderate-to-severe or severe (3 + ∕4 +) MR who met criteria for mitral valve surgery. Functional MR was present in 21% of patients; with the remainder having degenerative or combined degenerative and functional MR. Ninety percent of patients underwent successful clip placement with one third of the patients requiring placement of two clips. Acute procedural success, defined as placement of one or two clips with reduction of MR to <2+ occurred in 74% of patients with 66% were free from death, mitral valve surgery, or MR greater than 2+ at 12 months. The study demonstrated improvement in NYHA class III/IV symptoms from 55% to 8% at the end of 1 year. These outcomes were similar for both degenerative and functional MR. About 30% of patients required surgical intervention after clip placement at 3-year follow up with 84% success rate. The major adverse event rate was 9% with blood transfusions representing the majority of these events. No procedural mortality was encountered. Partial clip detachment occurred in 9% of patients, detected incidentally on 30-day echocardiography. All these patients were surgically treated successfully.

Following the encouraging results of EVEREST I study, the MitraClip device was subsequently compared with surgical mitral valve repair or replacement in the EVEREST II randomized-controlled trial^6^. The primary efficacy end point was freedom from death of any cause, surgery for MV dysfunction, and ≥3+ MR at 12 months. The primary safety end point was a composite of major adverse events within 30 days. The primary composite end point for efficacy was more frequent in the surgery group (73 versus 55%) due to higher rate of subsequent surgery for mitral valve dysfunction in the percutaneous group (20 versus 2%). Both approaches were associated with similar rates of overall mortality at one year (6% for both). Improvement in MR severity was seen in both groups with greater reduction in the surgical group. Presence of +3/+4 MR grade was similar at one year (21 versus 20%).

The five-year follow up results of the EVEREST II trial was recently published^12^. At five-year follow up, the primary efficacy end point in the as-treated population was reached in 44.2% in the percutaneous arm versus 64.3% in the surgical group (*p* = 0.01). The difference was driven by increased rates of 3+ to 4 + MR (12.3% vs. 1.8%; p = 0.02) and surgery (27.9% vs. 8.9%; p = 0.003) with percutaneous repair. The majority of surgical repairs after device therapy were encountered in the first 6 months (78% of surgeries); beyond 6 months, the rates of surgery and moderate-to-severe MR were comparable between groups. Similar rates of death in the percutaneous repair and surgical groups were reported at 5 years of follow up (20.8% vs. 26.8%, p = 0.4).

Left ventricular volumes and dimensions were significantly reduced from baseline in the two study groups with greater magnitude of reduction in the surgical group. At 5 year follow up, left ventricular dimensions were similar in both groups except for smaller left ventricular end-diastolic diameter in the surgical group (3.3 ± 0.9 cm vs 3.6 ± 0.9 cm, *P* < 0.009). Similar improvements in functional and quality of life were demonstrated at 12 months in both groups which persisted at 5 years follow up.

From the primary safety endpoint, major adverse event rates at 12 months were significantly lower in the MitraClip group compared to the surgical group (9.6 versus 57 percent). This was largely due to the higher rate of blood transfusion in the surgical group (8.8 versus 53.2 percent). The superiority of MitraClip with regard to safety end points persisted after adjusting for transfusion-related adverse events (0.7 vs 16.5%).

It is important to note that both EVEREST I and II trials included patients considered at low and moderate surgical risk. High surgical risk patients were subsequently evaluated in the EVEREST II High-Risk registry which included 78 patients with elevated surgical risk with predicted mortality greater than 12% (measured by the Society of Thoracic Surgery (STS) calculator)^13^. All patients with LVEF ≤20%, unfavorable MV anatomy, LV end-systolic diameter >60 mm, and MV area <4 cm2 were excluded from the study. The observed mortality in patients with MitraClip was 7.7% at 30 days as compared to mean predicted mortality of 18.2% by STS score. Compared to matched high-risk controls who were treated medically, there was improved 1-year survival in addition to improvement in NYHA functional class, favorable ventricular remodeling and reduction in the hospitalizations for heart failure as compared to baseline. The favorable safety profile of the MitraClip eventually led to FDA approval in October 2013 for the reduction of “symptomatic MR >3+ due to primary abnormality of the mitral apparatus (degenerative MR) in patients who have been determined to be at prohibitive risk for MV surgery by a heart team”.

## COAPT trial

Although it has been used successfully in functional mitral regurgitation, the role of MitraClip remains controversial in this population due to a lack of randomized data regarding the additive benefit of mitral valve repair in addition to optimal medical therapy. The COAPT trial, which is underway in multiple sites, will be the first randomized controlled trial to evaluate the potential benefit of MitraClip therapy in patients with heart failure and secondary MR who are at high surgical risk as compared to medical standard of care. Eligible candidates should be symptomatic with NYHA class 2 or greater and have grade 3 + ∕4 + MR due to cardiomyopathy of either ischemic or non-ischemic etiology. Patients should be considered high risk with predicted surgical mortality greater than 8% for inclusion. Primary endpoints include effectiveness at 1 year, defined as patients without recurrent heart failure hospitalizations, and a primary safety endpoint defined as freedom from all-cause mortality, stroke, worsening kidney function, and ventricular-assist device implantation or cardiac transplantation.

## Summary

Percutaneous mitral valve repair is an exciting new field with many devices at early developmental stages. The MitraClip repair system is the most advanced technique with the highest safety and efficacy to date, providing valuable alternative option to surgical intervention in patients with high or prohibitive surgical risk with degenerative mitral regurgitation. The role of the MitraClip in treating patients with functional mitral regurgitation remains unclear. The medical community is eagerly waiting for the results of the COAPT trial with hopes that it will lead to an expanded indication in the near future.
